# The Microvascular Anastomotic Coupler for Venous Anastomosis in Head and Neck Reconstruction: An Institutional Experience

**DOI:** 10.7759/cureus.64467

**Published:** 2024-07-13

**Authors:** Soroush Farsi, Michael Held, Madalyn Brannon, Peter Eckard, Deanne King, Emre Vural, Jumin Sunde, Mauricio Moreno

**Affiliations:** 1 Otolaryngology - Head and Neck Surgery, University of Arkansas for Medical Sciences, Little Rock, USA

**Keywords:** outcomes, predictors, venous coupler, vascular coupler, anastomosis, free tissue transfer, free flap, microvascular

## Abstract

Background

This study aimed to identify outcome predictors with the GEM microvascular coupler system (GEM Coupler) in a series of patients undergoing free flap reconstruction for head and neck defects.

Methodology

In this retrospective chart review of 218 consecutive microvascular procedures performed on 204 patients at an academic tertiary care center, demographics, comorbidities, surgical data, and outcomes were retrieved. The endpoints for the analysis were microvascular revision surgery and flap survival.

Results

The study included 142 (70.2%) males and 62 (29.8%) females, with a mean age of 56 years, primarily treated for malignancy (76%). The anterolateral thigh and fibula were the most commonly used flaps (40.4% and 27.1%, respectively). In 21 (9.6%) cases, a double venous anastomosis was performed. There were nine flap failures requiring microvascular revision surgery; the flap was salvaged in four of these cases yielding an overall success rate of 97.7%. Factors associated with total flap loss included a history of a thrombotic or embolic event (p = 0.017), deep circumflex iliac artery flap (p < 0.001), and absence of monitoring skin paddle (p < 0.001).

Conclusions

Prothrombotic conditions, buried flaps, and flap type are outcome predictors in patients undergoing microvascular reconstruction with GEM Coupler.

## Introduction

Microvascular free tissue transfer has significantly impacted the ability to resect large head and neck tumors while preserving an acceptable quality of life. Technical advances in the field of microsurgery have led to higher success rates, shorter operative times, and reduced complication rates [1].

Before the availability of mechanical anastomotic devices, hand-sewn venous microanastomoses were an absolute necessity. While still part of the standard of care, this approach may pose challenges, such as improper eversion of the vessel wall, endoluminal exposure of adventitia, and suture back-walling, and has been associated with increased operative times [2,3]. Overall, this is a highly technical and time-consuming portion of the procedure that is uniquely surgeon-dependent [4]. Mechanical anastomotic coupler devices are part of technical advances aimed at reducing interoperator variability and improving surgical outcomes. Since its initial description by Nakayama in 1962, the mechanical anastomotic coupler device has undergone evolution aimed at enhancing the patency of venous anastomosis and ensuring the reproducibility of results. The mechanical anastomotic coupler device offers several advantages over the hand-sewn anastomosis, including superior vessel wall eversion and intima contact, the rigid external stenting effect that mitigates vessel spasm, reduced likelihood of intima tears, and the absence of a foreign body within the vessel lumen [5]. The GEM Microvascular Anastomotic Coupler Device® (GEM Coupler) has been shown to be a safe and effective alternative to hand-sewn techniques for venous anastomoses, with the failure rate reported to be as low as 0.38% [1,2]. This device can also be utilized in arteries, as proven in a recent series of 50 patients where it was used successfully to perform all arterial and venous anastomoses without notable failures [4].

Within the last decades, free tissue transfer has become both widely available and an increasingly common option for the management of extensive head and neck defects. With this increased workload, microvascular surgeons are under constant pressure to reduce flap failures, perioperative morbidity, operative times, and costs. Globally, the documented failure rate for all microvascular free flaps is around 3-5% [6], but the failure rate with the use of the GEM Coupler has been reported to be around 3% by multiple studies [2,7]. Despite this, most studies have not been able to consistently identify predictors for flap failure or the need for surgical revision, which is at least in part related to the rarity of these events. As such, the limitations of the data regarding venous couplers and their utility in head and neck reconstruction remain, and there continues to be a lack of reliable outcome predictors throughout the medical literature. Even more, the heterogeneity of indications, practice patterns, and surgeon’s backgrounds further complicate the analysis and interpretation of the available data.

While coupler devices have demonstrated their effectiveness as an alternative to the hand-sewn technique, delivering favorable outcomes and contributing to an overall cost reduction in procedures, there is a scarcity of studies directly comparing both methods in the context of head and neck reconstruction. The present study aims to fill this gap by documenting microvascular outcomes specifically in head and neck reconstruction done by a single surgeon using the GEM Coupler. Furthermore, the study seeks to identify predictors for revision surgery and flap survival within this contemporary series.

## Materials and methods

This study was conducted at the University of Arkansas for Medical Sciences (UAMS), a tertiary academic center in Little Rock, Arkansas, United States. The study design received approval from the university’s Institutional Review Board (IRB# 228942). The patient database of the Department of Otolaryngology - Head and Neck Surgery was queried to identify all patients who underwent a microvascular reconstructive procedure within a four-year period. The inclusion criteria for the study were defined as (1) all venous microanastomoses performed with the GEM Coupler; (2) the reconstructive procedure was performed at a UAMS-affiliated hospital; (3) all indications for surgery were included (oncologic and non-oncologic); (4) the procedure-related documentation was available for review; and (5) a minimum postoperative follow-up of two months. The exclusion criteria were defined as (1) any venous microanastomosis performed with the hand-sewn technique; (2) microvascular anastomosis in the context of tissue replantation; (3) procedure performed at a non-affiliated hospital; and (4) inadequate documentation or follow-up.

Of the 209 patients originally considered for the study, five cases were excluded for the following reasons: hand-sewn anastomoses in four cases, and microsurgery performed for facial replantation in one case, leaving a cohort of 204 patients. The charts, operative reports, intraoperative photographs, and hospital documentation of these patients were reviewed. The information retrieved included demographics, comorbidities, smoking status, surgical data, and flap outcomes.

Microvascular variables included flap type(s), ischemia time(s), recipient vessel(s), coupler size(s), and the use of implantable Cook-Swartz Doppler Flow Monitoring System® (Cook Vascular Inc., Vandergrift, PA). At our institution, this device is used exclusively in the absence of an external skin paddle for monitoring and denotes the presence of a buried flap. When a double venous anastomosis was performed, the larger coupler was defined as primary while the smaller device was considered as secondary. In these cases, the total coupler area was calculated by adding the diameters of both couplers. For patients who underwent reconstruction with more than one free flap, synchronically or otherwise, all data was collected and analyzed for each flap. Any postoperative revision or surgical intervention was recorded up to 30 days postoperatively. In cases that underwent microvascular revision, arterial and venous findings and their management were documented independently.

Technical considerations and routine patient care

All procedures were performed by the senior author (M.M.) under surgical microscope magnification. Immediately after harvesting, all flaps were irrigated with heparin solution (50 U.S.P. Heparin Units/mL N.S.) and routinely inset under ischemia. The mechanical anastomotic device used in all cases was the GEM Microvascular Anastomotic Coupler Device® (Synovis Micro Companies Alliance, Birmingham, AL), which was used according to the manufacturer’s recommendations. All venous anastomoses were performed in an end-to-end fashion.

At our institution, routine patient care included overnight observation in intensive or intermediate care units, followed by observation in a dedicated surgical floor until discharge, between postoperative days (PODs) five and seven. Clinical flap monitoring, performed exclusively by the nursing staff, occurred every hour for the first 48 hours, every two hours for the following 48 hours, and subsequently every four hours until discharge. For routine thromboprophylaxis, lower extremity sequential compression devices were used, along with enoxaparin 40 mg administered subcutaneously starting on POD one.

Statistical analysis

The statistical analysis was performed with SPSS Statistics® version 20 (IBM Corp., Armonk, NY, USA). The information retrieved from the patient database was in the form of tabulated data. Categorical patient characteristics were summarized as proportions while continuous characteristics were summarized as medians and quartiles. Clinical endpoints for the analysis were microvascular surgical revision and total flap loss. Student’s t-test and Pearson’s chi-square tests were used to establish the association between continuous and categorical variables, respectively. All analyses were univariate and p-values <0.05 were considered statistically significant.

## Results

A total of 218 free flap procedures were performed on 204 patients. The cohort comprised 142 (70.2%) males and 62 (29.8%) females, with a mean age of 56.2 years (range = 2 to 87). The indications for reconstruction are summarized in Table 1. The most common indication was malignancy in 76% of the cases, with benign tumors and trauma/chronic wounds each accounting for 7.3% of the cohort.

**Table 1:** Summary of demographic characteristics of the cohort and indications for reconstruction (n = 204).

	Number	%
Age	Mean = 56.2	Range = 2–87 years
Sex		
Male	142	69.6%
Female	62	30.4%
Indication		
Malignancy	157	76%
Benign disease	15	7.3%
Trauma/Chronic wound	15	7.3%
Osteoradionecrosis	12	5.9%
Arteriovenous malformation	6	2.9%

Table 2 presents a summary of medical comorbidities and radiation exposure. Overall, hypertension was the most prevalent comorbidity (27.1%), followed by coronary artery disease (9.2%) and diabetes (7.8%). Six patients (2.8%) had a history of an embolic episode and/or prothrombotic disorder, including deep vein thrombosis (DVT), pulmonary embolism (PE), or factor V Leiden. In total, 24 (11%) patients had a history of head and neck radiation.

**Table 2:** Medical comorbidities and exposure to radiation (n = 204).

Comorbidities	Number	%
Kidney disease	4	1.8%
Arrhythmia	6	2.8%
Hypertension	59	27.1%
Diabetes	17	7.8%
Coronary artery disease	20	9.2%
Chronic obstructive pulmonary disease	14	6.4%
Hypothyroidism	12	5.5%
Thrombotic/Embolic history	6	2.8%
Hyperlipidemia	13	6%
Autoimmune disease	7	3.2%
History of XRT	24	11%

The defects were grouped into major categories, which are presented in Table 3. The most common defect location was the oral cavity (46.3%), followed by cutaneous/soft tissue defects (17%) and orbitomaxillary defects (13%). In total, 13 patients underwent reconstruction with multiple flaps (triple flaps in one case); of these, seven cases were performed synchronically while six were either staged or delayed. The most common flaps performed were the anterolateral thigh in 40.4%, followed by the fibula in 27.1%, and the fasciocutaneous radial forearm flap in 17.9% (Table 4).

**Table 3:** Location of the primary defect (n = 204).

Defect site	Number	%
Orbitomaxillary	29	13%
Oral cavity	101	46.3%
Oropharynx	17	7.8%
Palate	25	11.5%
Cutaneous/Soft tissue	37	17%
Scalp	9	4.1%

**Table 4:** Free flaps used for reconstruction (n = 218).

Flap	Number	%
Anterolateral thigh	88	40.4%
Anteromedial thigh	1	0.5%
Deep circumflex iliac artery	4	1.8%
Fibula	59	27.1%
Latissimus dorsi	7	3.2%
Rectus abdominis	5	2.3%
Fasciocutaneous radial forearm	39	17.9%
Osteocutaneous radial forearm	2	0.9%
Scapula/Scapular tip	5	2.3%
Temporoparietal fascia	1	0.5%
Vastus lateralis	7	3.2%

All arterial anastomoses were hand-sewn with interrupted 9-0 or 10-0 nylon, and the facial and lingual arteries accounted for more than 80% of all recipient vessels (Table 5). A single venous anastomosis was performed in 197 (90.4%) patients while a double anastomosis was performed in 21 (9.6%) patients. The most commonly used recipient vein was the facial vein in 151 (69%) cases, followed by the external jugular vein in 24 (11%) cases (Table 6). The information regarding the coupler sizes is summarized in Table 7. We found that the 3 mm coupler was the most frequently used device for primary anastomoses (29.4%), while the 2.5 mm device was the most commonly used for secondary venous outflow (6.9%).

**Table 5:** Recipient artery used for microanastomosis (n = 218).

Recipient artery	Number	%
External carotid	4	1.8%
Facial	139	63.8%
Lingual	37	17%
Occipital	1	0.5%
Superior thyroid	11	5%
Superficial temporal	13	6%
Transverse cervical	13	6%

**Table 6:** Recipient vein used for microanastomosis (n = 218).

Recipient vein	Number	%
Anterior jugular	7	3.2%
Peripheral branch	13	6%
External jugular	24	11%
Facial	151	69%
Retromandibular	4	1.8%
Superficial temporal	12	5.5%
Transcervical	7	3.2%

**Table 7:** Number of venous anastomoses and coupler sizes (n = 239).

	Number	%
Number of venous anastomoses		
Single	197	90.4%
Double	21	9.6%
Primary coupler size		
1.5 mm	2	0.9%
2.0 mm	11	5%
2.5 mm	47	21.6%
3.0 mm	64	29.4%
3.5 mm	55	25.2%
4.0 mm	39	17.9%
Secondary coupler size		
1.5 mm	1	0.5%
2.0 mm	3	1.4%
2.5 mm	15	6.9%
3.0 mm	2	0.9%

Within the first 30 postoperative days, 24 cases required surgical revision for the following reasons: six cases due to wound dehiscence, four cases due to hematoma, four cases due to tissue debridement, and three cases due to infection. In nine (4.1%) of these cases, there was a microvascular complication that required surgical exploration. Most of these (n = 5, 56%) presented in association with other surgical complications such as hematoma or infection, while the remaining cases (n = 4, 44%) were purely microvascular. Overall, there were four arterial failures (three thromboses and one vessel kink), four venous failures (three thromboses and one external compression), and one combined arterial/venous thrombosis, yielding an overall coupler-related failure of 2.3%. Salvage microanastomosis was attempted in all of these cases, and it was successful in four (44.5%), yielding an overall flap success rate of 97.7%. All microvascular failures occurred during the first postoperative week. Partial flap loss was observed in two cases (15% and 20%), and it was managed conservatively.

Table 8 summarizes the statistical correlation between clinical and surgical variables and microvascular outcomes. In terms of defect location, orbitomaxillary defects were associated with a higher rate of microvascular revision (11.5% vs. 4.1%, p = 0.045). Predictors for total flap loss included a history of a thromboembolic event or prothrombotic condition and reconstruction with deep circumflex iliac artery (DCIA) flap (p = 0.017 and p < 0.001, respectively). The absence of a monitoring skin paddle was the only variable associated with both microvascular revision and flap loss (p = 0.033 and p < 0.001, respectively). The average ischemia time was 118 minutes (range = 50-300 minutes), and it was not a predictor for surgical re-exploration (p = 0.981) or flap outcomes (p = 0.874).

**Table 8:** Summary of p-values for correlation between variables and observed outcomes. Statistically significant associations are highlighted. §: Includes the need for microvascular revision and/or partial flap loss. †: Considers the combined sizes of both devices in patients with double anastomosis. a: Pearson’s chi-square test; b: Student’s t-test.

	Microvascular complication^§^	Total flap loss
Comorbidities and patient’s factors		
Renal disease	0.626^a^	0.757^a^
Arrhythmia	0.549^a^	0.704^a^
Hypertension	0.868^a^	0.719^a^
Diabetes	0.943^a^	0.511^a^
Coronary artery disease	0.257^a^	0.472^a^
Chronic obstructive pulmonary disease	0.351^a^	0.553^a^
Hypothyroidism	0.39^a^	0.585^a^
Prothrombotic disorder	0.224^a^	0.017^a^
Hyperlipidemia	0.37^a^	0.569^a^
Radiation therapy	0.21^a^	0.426^a^
Autoimmune disease	0.516^a^	0.68^a^
Smoking	0.239^a^	0.303^a^
Surgical variables		
Defect location	0.045^a^	0.061^a^
Flap type	0.06^a^	p < 0.001^a^
Recipient artery	0.786^a^	0.319^a^
Double venous anastomosis	0.245^a^	0.46^a^
Primary coupler size	0.75^a^	0.661^a^
Total coupler area^†^	0.835^b^	0.71^b^
Anastomosis to the superficial venous system	0.356^a^	0.923^a^
Buried flap (no monitoring skin paddle)	0.033^a^	p < 0.001^a^
Total ischemia time	0.981^b^	0.874^b^

## Discussion

Free tissue transfer is an increasingly popular option for the management of complex or extensive head and neck defects. This field is constantly evolving, with new technical and technological innovations aimed at expanding its indications and improving surgical outcomes. Since its conceptual inception in the late 1980s, the GEM Coupler has been a well-received addition to the microvascular surgeon’s armamentarium, and it is widely regarded as an effective alternative to hand-sewn anastomosis. The GEM Coupler has been shown to decrease ischemia and operative times, while at the same time reducing some technical risks of the hand-sewn technique, such as endoluminal inclusion of adventitia and back-walling [2]. The incidence of total flap failure in the literature is routinely described as 3-5% [8,9]. In this regard, the failure rate of 2.3% observed in the present cohort compares favorably to most published series [6].

The coupler patency rate of our study was 98.2%, with only four flaps requiring venous anastomotic revision. This is in agreement with other studies that consistently report higher patency rates with this device versus hand-sewn techniques [7,10-12]. The GEM Coupler has also been found to tolerate higher tension and axial loading than sutured anastomoses within the first two postoperative weeks [11]. In a series of 151 anastomoses performed with the device, Yap et al. reported a patency rate of 98.7% which is consistent with our findings [12]. Overall, our study confirms and supports the safety and efficacy of the microvascular coupler system in the setting of head and neck reconstruction.

Given the low failure rate and limited number of patients, most series have failed to identify reliable outcome predictors for patients undergoing microvascular reconstruction with a GEM Coupler in the field of head and neck surgery. In this regard, the present communication differs from most published series, as we were able to establish predictors for both microvascular revision and flap failures.

Among all of the patient-related factors analyzed, we found that a history of an embolic event or prothrombotic condition (DVT, PE, factor V Leiden) was associated with higher flap failures (p = 0.017), but not with microvascular compromise amenable to revision. This suggests that prothrombotic states yield more severe complications and render flap salvage less likely in an acute event. This notion is supported by a recent study by Wang et al., who reviewed the outcomes of 2,042 flaps in hypercoagulable patients and reported a complete flap loss rate of 15.5% with a salvage rate of 0% [13]. Their definition of hypercoagulable states included all of the conditions described in the present series, and, similarly, our study mirrors the 0% salvage rate for these individuals. These findings suggest that in the presence of prothrombotic states, microvascular outcomes are primarily governed by altered physiological mechanisms, and seem to be relatively independent from technical considerations.

The type of flap utilized was also a predictor for total loss (p < 0.001), with two cases found among DCIA flaps. One patient experienced a venous thrombosis on POD zero and it could not be salvaged despite an early reintervention and aggressive use of tissue plasminogen activator. The other failure presented with a combined arterial and venous thrombosis POD three. The venous thrombosis was distal to the coupler so it is presumed that the coupler did not contribute to the failure. Neither of these patients had significant comorbidities or past medical history, except for one who smoked up until surgery. As osteotomies were performed in both of these cases and both of them were buried, we question if additional technical considerations may have played a role in the genesis of these outcomes.

The location of the defect was a predictor for microvascular revision, with orbitomaxillary defects having a higher complication rate than all other sites (11.5% vs. 4.1%, p = 0.045). Interestingly, this difference did not translate into a higher flap failure rate. We hypothesize that this might reflect the increased technical demands that characterize midface reconstruction, particularly when compared to more common and straightforward defects. Among other challenges, midface reconstruction is characterized by a lack of adjacent recipient vessels, complex three-dimensional anatomy, exposure to flora from both oral and nasal cavities, and risk for inadequate drainage of accumulated secretions or collections. As such, it is conceivable that these patients are at an inherently elevated risk for complications.

Of all the variables tested, the lack of a monitoring skin paddle (buried flap) was the only one that proved to be a significant predictor for both microvascular revision and flap failure. In the literature, there is still controversy regarding the impact of buried flaps on microvascular outcomes. Lindau et al. reviewed the outcomes of 103 buried flaps for head and neck reconstruction. They reported a 4.9% complication rate requiring reoperative intervention, but there were no flap losses within the first two postoperative weeks. The authors concluded that there were no differences in flap failure rates between buried flaps and externally monitored flaps [14]. Conversely, in a series of 49 flap failures from a total of 1,310 surgeries performed at M.D. Anderson Cancer Center, Yu et al. found that buried flaps were the most common cause of delayed flap failure [15]. Our findings support this observation. Overall, while the implantable Doppler has a definitive role in the microvascular armamentarium, it must be recognized that its use does not improve salvage rates [16] and that clinical monitoring is still considered the standard of care [17].

While our study did not include an economic analysis, several authors have conducted such analyses on microvascular coupler usage, highlighting a notable reduction in anastomosis time and, consequently, overall operation theater utility time. On average, these studies revealed savings of £154 and $234.89 per coupler usage [18,19]. In contrast, Senthil Murugan et al. found that the cost of couplers was not justified despite the reduction in operation theater utility time. Nevertheless, all three studies underscored the cost-effectiveness of coupler devices, especially in situations where they contributed to reducing return-to-operation theater rates by significantly lowering the incidence of venous thrombosis [20].

We found no association between ischemia times and microvascular outcomes. While recent data is scarce in this regard, ischemia times under five hours have shown to have a negligible effect on anastomotic complications in patients undergoing reconstruction with a free fibula flap [21]. Our average ischemia times fall well within this time frame, which might explain the lack of association with microvascular outcomes.

While the study provides valuable insights, it is important to acknowledge certain limitations. The retrospective design inherently restricts the ability to establish causation or control for all potential confounding variables. The reliability of the study’s findings depends on the accuracy and completeness of available medical records, with variations in documentation practices over time potentially introducing inconsistencies. Additionally, the outcomes may be influenced by the specific patient population and surgical techniques employed at the single center, raising concerns about the generalizability of the results to broader clinical settings. Therefore, caution is advised in extrapolating the findings, and future multi-center prospective studies are needed to validate and expand upon these observations in diverse clinical contexts.

## Conclusions

Anastomotic coupling devices have proven to be safe in the literature and that remains true in our patient population. We found that a history of prothrombotic conditions, buried flaps, and DCIA flaps were associated with higher failure rates. The findings presented herein, along with other relevant considerations, should be taken into account when planning and executing free tissue transfer procedures for head and neck defects. Further studies are necessary to validate these findings across institutions and to help elucidate additional factors affecting microvascular outcomes in this setting.
